# "Brace Technology" Thematic Series - The Lyon approach to the conservative treatment of scoliosis

**DOI:** 10.1186/1748-7161-6-4

**Published:** 2011-03-20

**Authors:** Jean Claude de Mauroy, Cyril Lecante, Frédéric Barral

**Affiliations:** 1 Clinique du Parc, 155 boulevard Stalingrad 69006 Lyon, France; 2 Groupe Lecante, 125 rue Bataille 69007 Lyon, France

## Abstract

The Lyon Brace, or adjustable multi-shell brace, has been used for more than 60 years.

The use and function of the Lyon Brace includes:

- The utilization of one or two corrective plaster casts, which enables a true lengthening of the concave ligaments.

- An oriented CAD-CAM moulding in 3D auto correction after the removal of the plaster cast.

- A blueprint adapted to Lenke's classification.

- A specific physiotherapy program.

**Background:**

Pierre Stagnara created the Lyon Brace in 1947. The brace has the following characteristics:

- It adjusts to allow for a child's growth of up to seven centimetres and for an increase in weight of up to seven kilograms.

- It is 'active' in that the rigidity of the PMM (polymetacrylate of methyl) structure stimulates the user to auto-correct. The active axial auto-correction decreases the pressures of the brace on the trunk.

- It is decompressive in that the effect of extension between the two pelvic and scapular girdles decreases the pressure on the intervertebral disc allowing for more effective pushes in the other planes.

- It is symmetrical making it both more aesthetically pleasing and easier to build.

- It is stable at both shoulders and pelvic girdle, facilitating the intermediate 3D corrections.

- It is transparent. The pressure of the shells on the skin can be directly controlled so "pads" are usually not necessary.

**Brace description:**

Two metal bars are fixed vertically, one anterior the other posterior and all shells are attached from the bottom to the top in this order:

- Two pelvic shells ensure an optimal stability of the brace.

- One lumbar shell T12-L4, which can be either independent or extending, at the abdominal chondrocostal level.

- One thoracic shell at the level of the thoracic convexity.

- One opposite thoracic shell used as a counter push.

- One shoulder balance shell on the side of the thoracic convexity.

**Long term follow up results:**

This is a retrospective study of 1,338 completed treatments checked a minimum of two years after weaning from the brace.

Only 5% of the curves progressed more than 5° from the initial magnitudes. This translates to an effectiveness index of 0.95.

A subset of 174 subjects who started treatment at Risser 0 was isolated. The global progressive angular mean curve was superimposed on the statistic general curve and the effectiveness index was calculated at 0.80.

The Surgery rate was just 2% of the patients presenting with an initial curve below 45°.

**Conclusion:**

The Lyon Brace is the historical reference of bracing AIS. To be fully effective, it requires the patient to wear a plaster cast for at least one month and receive specific physiotherapy training. Although this is a retrospective study, the results are very positive, and clearly indicate a need for a prospective study.

## Introduction

**Figure 1:**
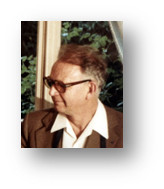
**Pierre Stagnara**. Original picture taken in 1985 by JCdM.

The Lyon Bracing Management has evolved since 1947 as a cooperative effort between Pierre Stagnara MD (Figure 1) and Bouillat & Terrier CPO [1]. The Lyon management protocol requires curve reduction with a plaster cast for one to four months, after which the brace is moulded and fit. The brace is a highly adjustable under the arm design and does not have a superstructure [2].

## History of the Lyon Brace

**Figure 2:**
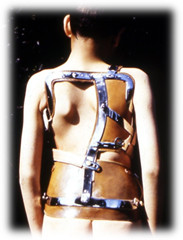
**Original historic Lyon brace**. Old Lyon Brace realized by Bouillat and Terrier.

Very early braces were made from a combination of steel and leather (Figure 2).

**Figure 3:**
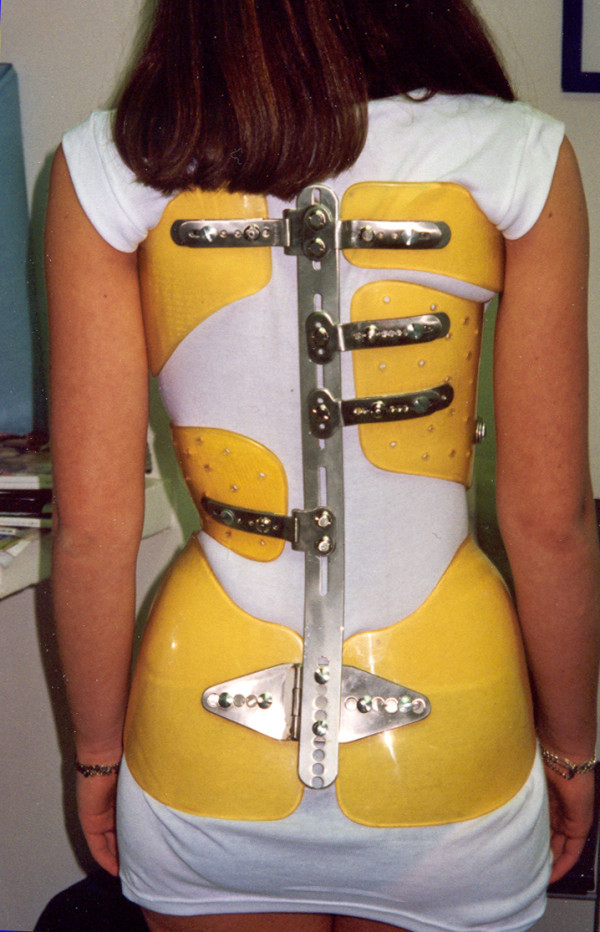
**Lyon brace - posterior view**.

**Figure 4:**
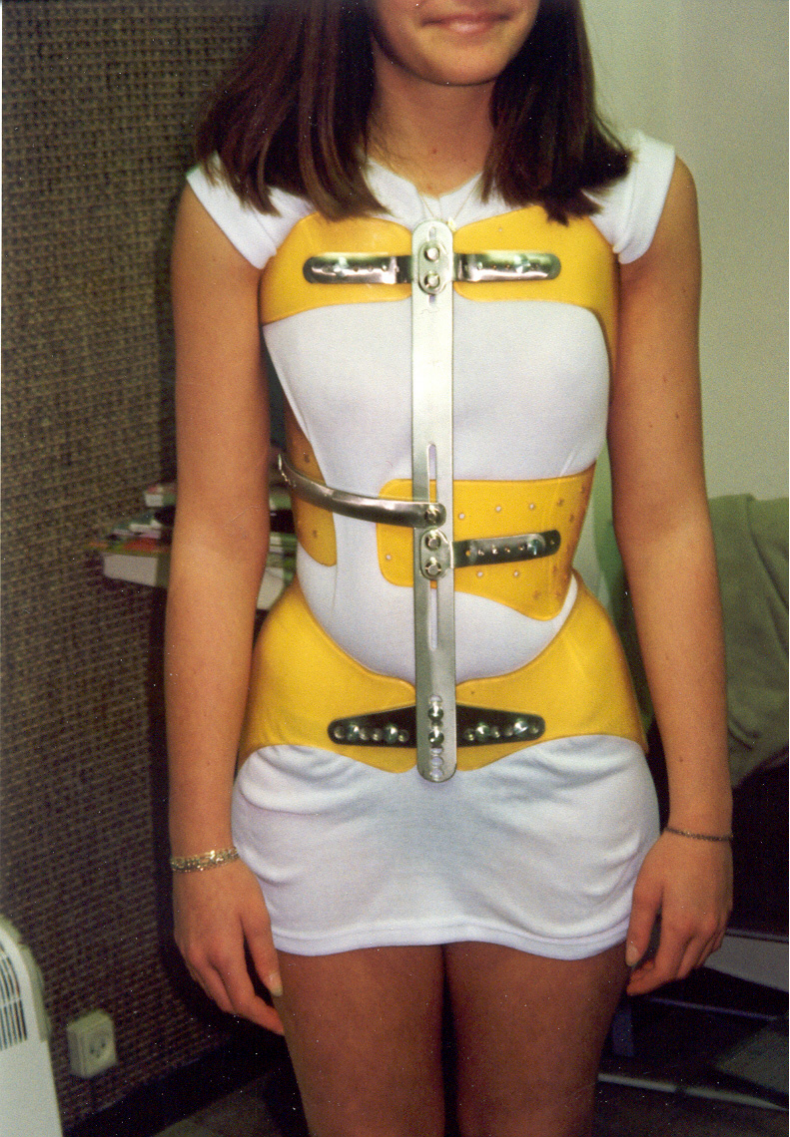
**Lyon brace - anterior view**.

Following World War II, Régis Lecante, CPO, discovered a Messerschmitt cockpit near his country house. Made of a very durable plastic, it could be easily machined, tapped and screwed. The use of PMM (polymetacrylate of methyl) or Plexidur™ was born, revolutionizing modern brace materials and design. (Figure 3, 4)

The Lyon Brace, created in 1947 by Pierre Stagnara, is designed to be:

- Adjustable: It is easily modified, accommodating up to 7 cm of growth, while also being more cost effective, requiring fewer and less frequent changes.

- Active: The rigidity of the PMM structure stimulates the child to initiate an active axial auto correction which decreases superficial pressures.

- Decompressive: As a consequence of the "Adjustable" feature, the effect of extension between the two pelvic and scapular girdles also decreases the pressure on the intervertebral discs and promotes more effective pushes.

- Symmetrical: In addition to being more aesthetically pleasing, the brace is much easier to build.

- Stable: The stability of both shoulder and pelvic girdle facilitates the intermediate corrections.

- Transparent: Skin pressures are easily observed and direct control of the pushes, stops, drives and reliefs is possible. Complementary pads are rarely needed.

**Figure 5:**
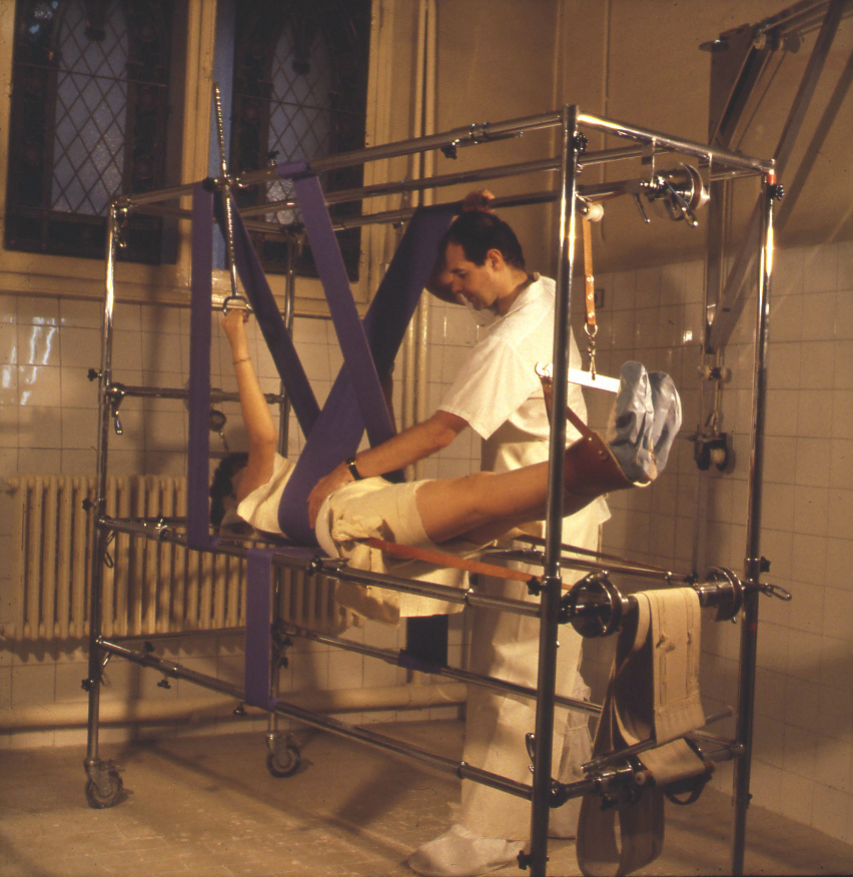
**Cotrel's EDF frame to apply the plaster cast**.

The Lyon Brace is always constructed after the completion of the reductive plaster casting protocol using a Cotrel EDF frame (Figure 5).

The plaster casting methodology has never been abandoned because a better solution has not been found [3].

The specialized physiotherapy begins while the patient is in the plaster cast and is continued during the period of bracing; it is a fundamental and essential part of the treatment.

## Theoretical principles

Ligaments can retain their changed shape when stretched past a certain point or for a prolonged period of time. The plaster cast gradually increases the length of the musculoligamentous structure of the concavity through continuous traction over a minimum of four weeks. The brace then maintains the viscoelastic level of the structure, especially at night.

When the curve is above 30°, the brace carries out an anti-collapsing function during the day.

This elongation diminishes the constraints on the disc and makes easier the 3D correction of the scoliosis.

In the frontal plane, the action is made of 3 points system.

**Figure 6:**
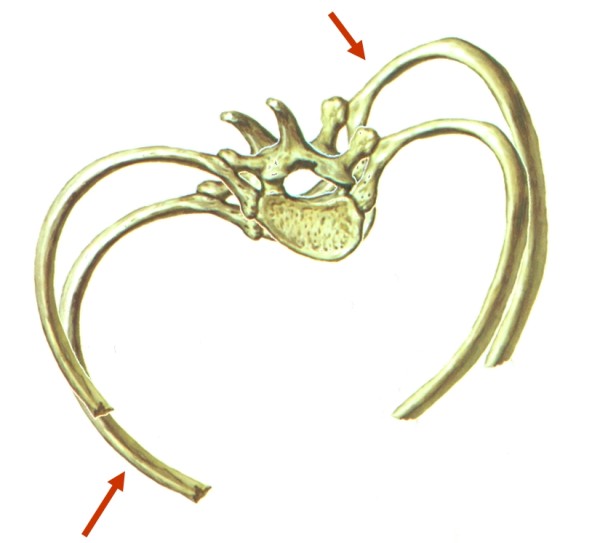
**Biomechanical effect of the brace in horizontal plane**.

The thoracic derotation is obtained with a push on the internal side of the rib hump and an anterior chondrocostal concave counter push. We avoid every postero-anterior push on the rib hump to not increase the flat back. (Figure 6)

At the lumbar level, we realize the push on a convex transverse.

**Figure 7:**
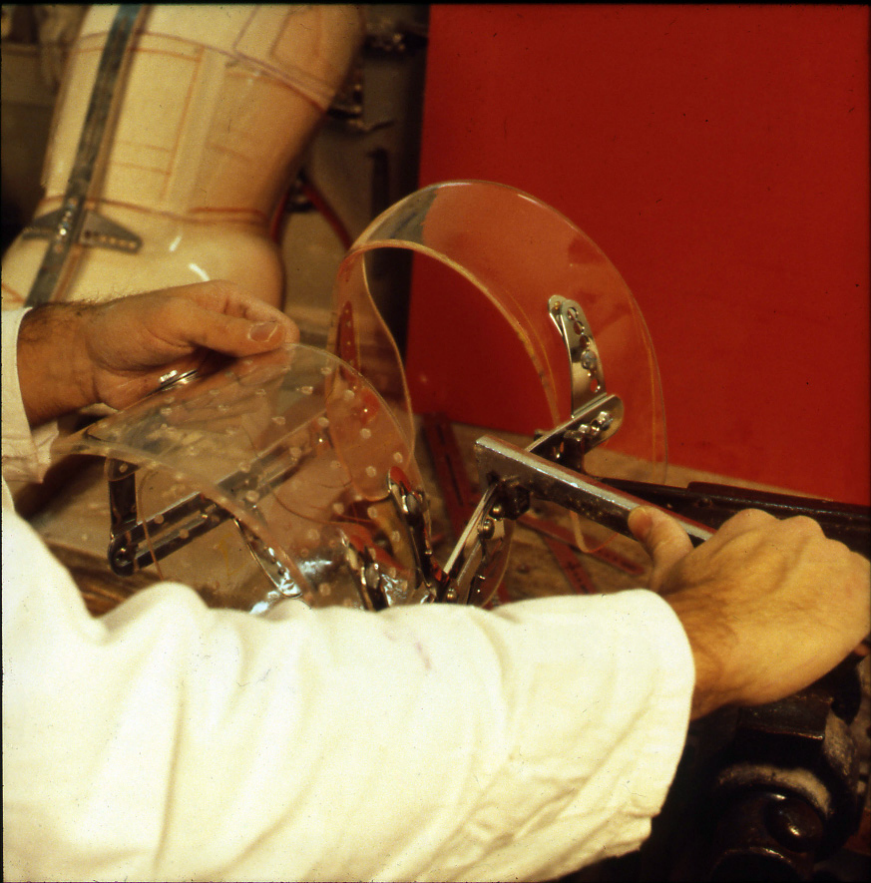
**Lordotization of the posterior bar in sagittal plane**.

In the sagittal plane, we accentuate the lumbar lordosis in order to increase the kyphosis of the thoracic region by sagittal bending of the bars. (Figure 7)

### Description of the brace

Different curves require different placement of pushes according to curve severity and location [3].

**Figure 8:**
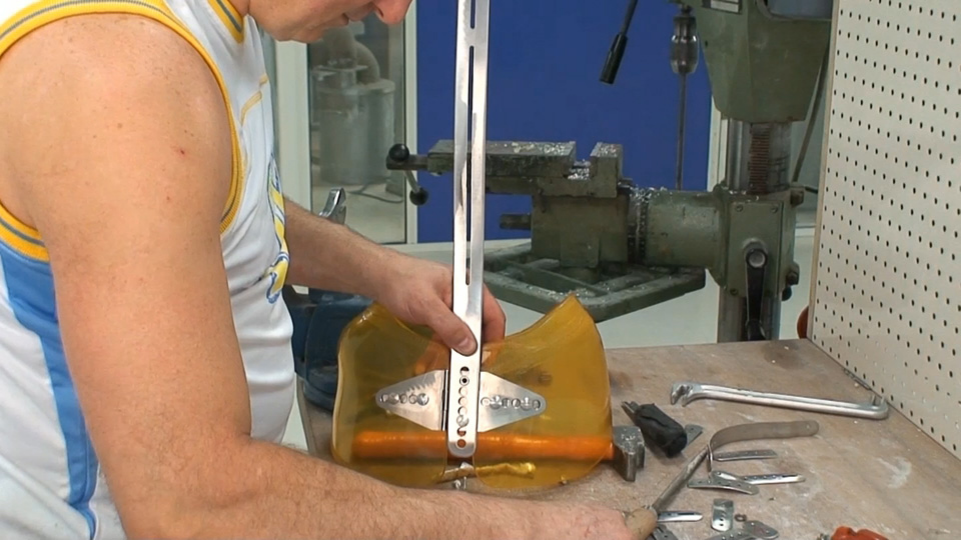
**Vertical posterior bar**.

**Figure 9:**
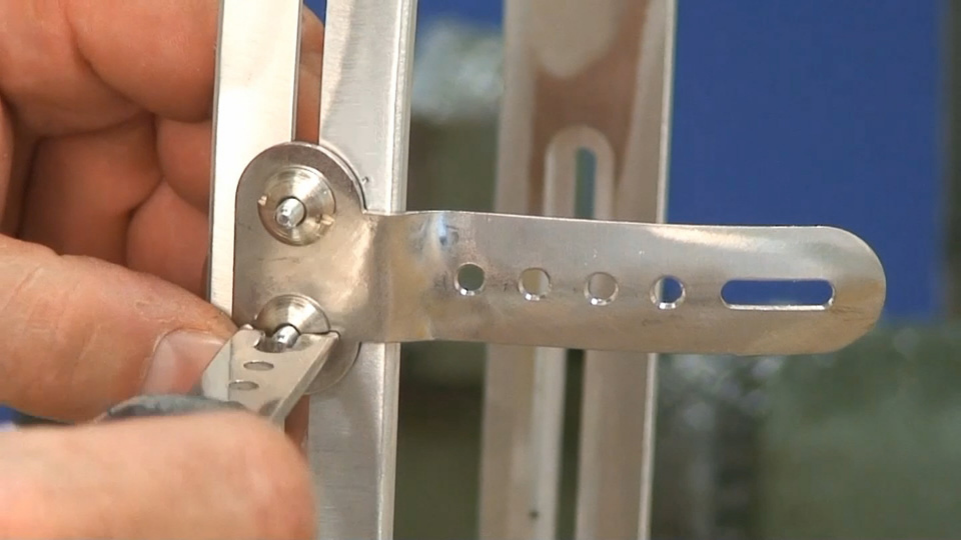
**Faceplate in high steel**.

**Figure 10:**
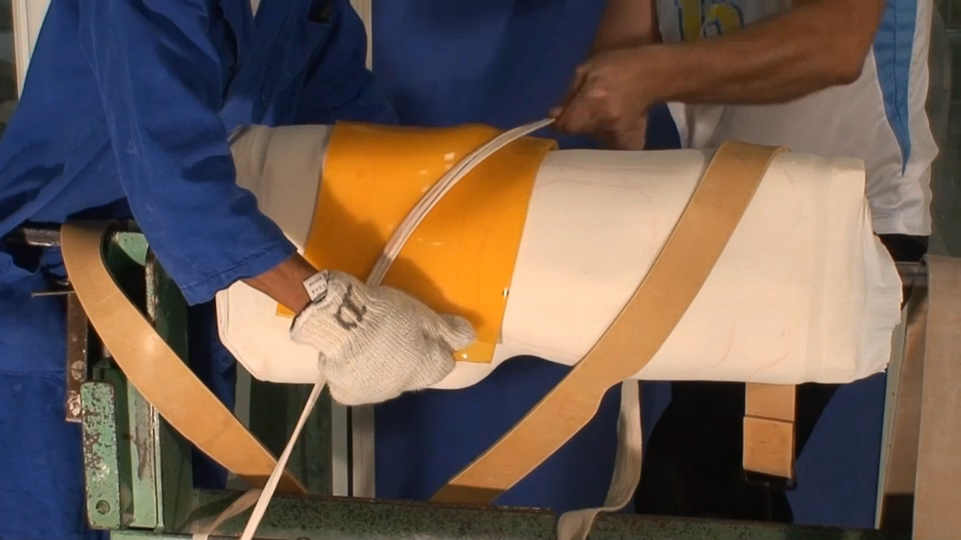
**Thermo malleable shells made in PMM**.

The bars are made of radio-transparent duralumin (Figure 8), the faceplate and joints of steel (Figure 9), and the thermo malleable plastic is made of PMM (Figure 10).

**Figure 11:**
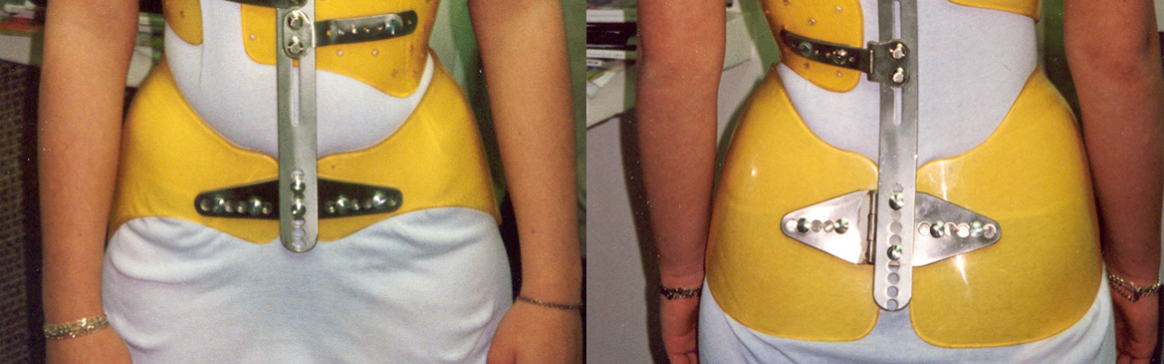
**Pelvic basis**.

**Figure 12:**
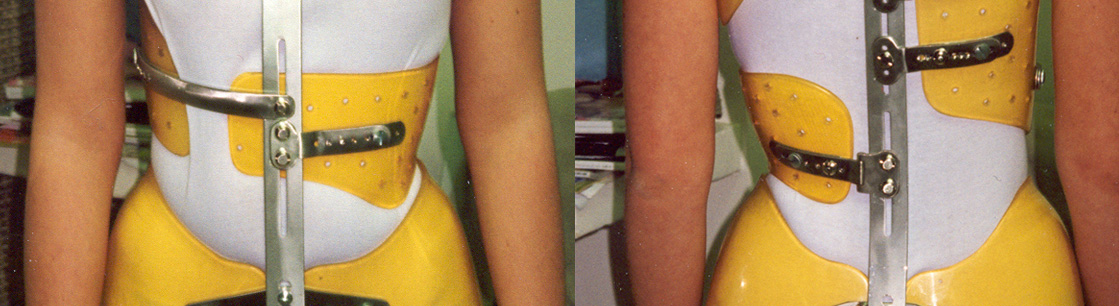
**Lumbar shell**.

**Figure 13:**
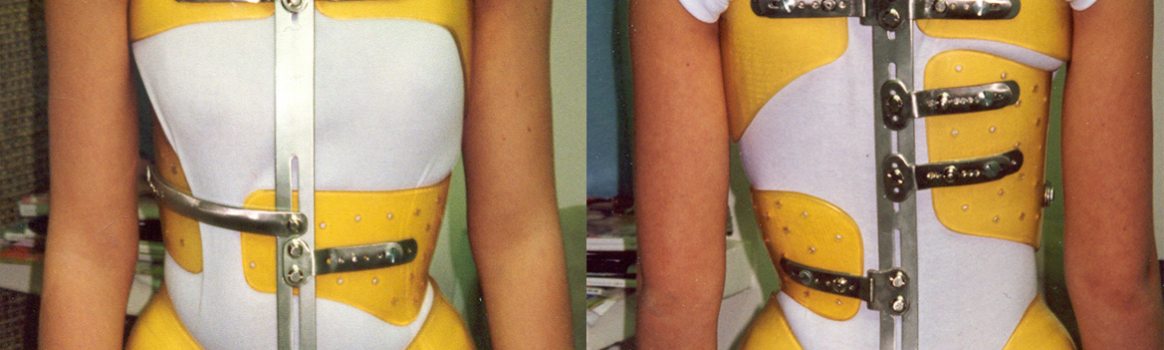
**Thoracic main shell**.

**Figure 14:**
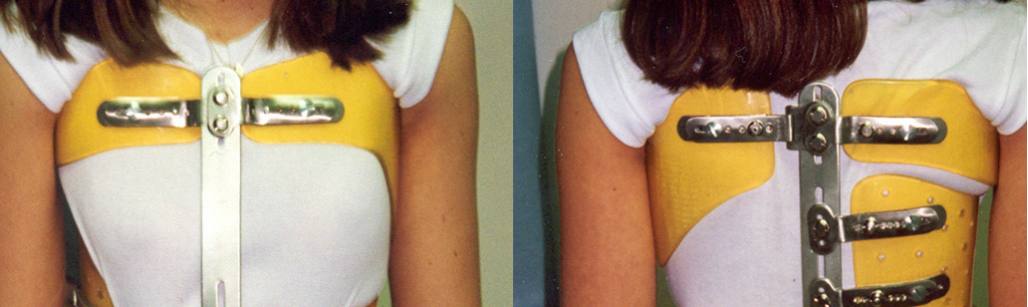
**Axillary counter push & opposite balance shell**.

The finished brace is comprised of:

- A pelvic section insuring optimal stability of the brace, (Figure 11).

- A lumbar shell covering levels T12-L4, either independent or extending at the abdominal chondrocostal level, (Figure 12).

- An abdominal or anterior shell that extends laterally. This is the case especially on the opposite side of the thoracic convexity which drives derotation of the anterior counter rib hump, extends in height to contain the abdomen, and barely covers the inferior margins of the ribs and xyphoid process like an abdominal apron. This shell is a crucial point for kyphotisation and derotation.

- A thoracic shell at the level of the convexity (Figure 13).

- An opposite thoracic shell used as a counter push (Figure 14).

**Figure 15:**
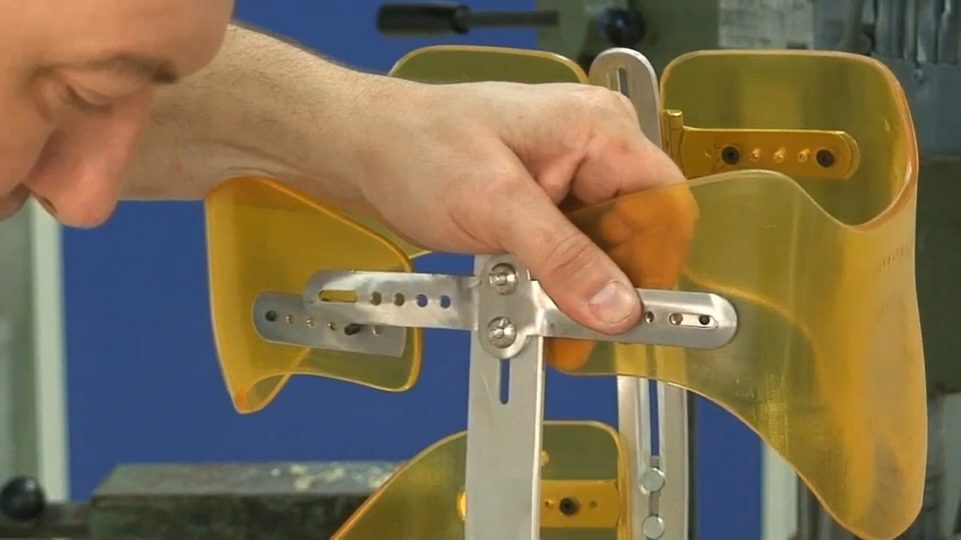
**Metallic plates**.

Eventually, a small axillary shell can be used to balance the shoulders on the side of the convexity. The shells are fixed on the two bars by metallic plates allowing some modifications with growth (Figure 15).

The PMM shells can provide a stop, a drive or a push, depending on the way they are applied.

### Three brace Types

**Figure 16:**
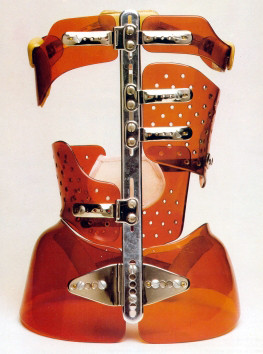
**Lyon thoracic & double major brace**.

1) The Lyon classical thoracic or double major brace is composed of:

- A pelvic girdle with two hemi shells and two posterior and anterior bars which allows for adjustments.

- A series of shells strategically applied to push forces at a medial thoracic level (T7-T12), a high thoracic level (T4-T7), a lumbar level (T12-L4), an anterior chondrocostal asymmetric approach, and eventually an axillary balancing scapular support (Figure 16).

2) The Lyon lumbar brace was created by Michel and Allègre [4].

**Figure 17:**
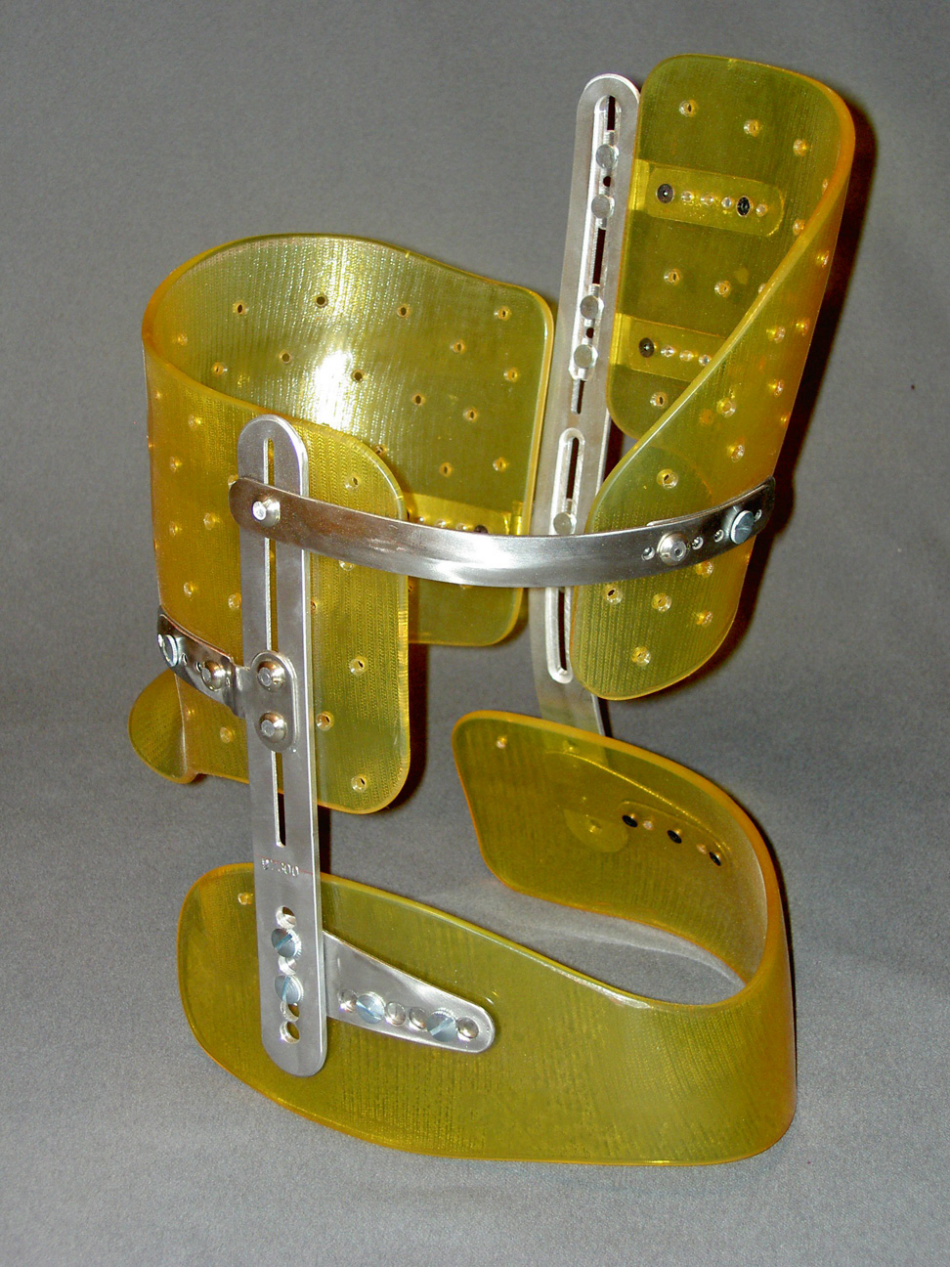
**Lyon lumbar brace**. or Three points brace of Michel & Allegre

**Figure 18:**
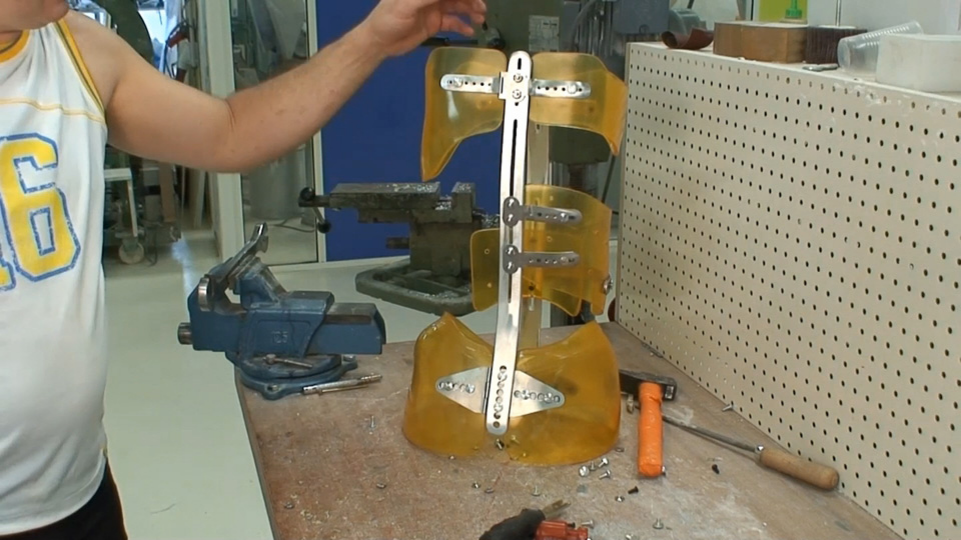
**Lyon thoraco-lumbar brace**.

There are three main components:

- An ilio-lumbar shell (T11-L4) on the convex side with a characteristic horizontal support on the iliac crest that elicits a reflex of axial extension.

- A trochanteric hemi circle on the concave side.

- A thoracic shell (T6-T12) on the concave side. (Figure 17)

3) The Lyon thoraco-lumbar brace is a 3 points high brace with:

- A Large thoraco-lumbar push (T6-L2) with no lumbar shell.

- A high thoracic push (T4-T7). The lever arm is maximal in the coronal plane. (Figure 18).

### Indications

The Lyon Management protocol follows the SRS guidelines for initiating bracing. The minimal curve indications are 20° and above during the period of accelerated growth for 11 to 13 year old girls, and 30° during diminishing pubertal growth. No preventive treatment is prescribed. In some cases, the treatment is anticipated when scoliosis has been previously diagnosed in other family members or when a significant rotation or imbalance of the occipital axis is observed. Sometimes a scoliosis of magnitude greater than 40° is braced when recommendations for surgery are refused by the parents.

The Lyon brace is particularly suitable for patients to wear during rapid pubertal growth, while the Milwaukee brace is better suited to pre pubertal juveniles because it is less likely to cause rib-cage deformity. It is used whatever the aetiology in ambulatory children.

**Figure 19:**
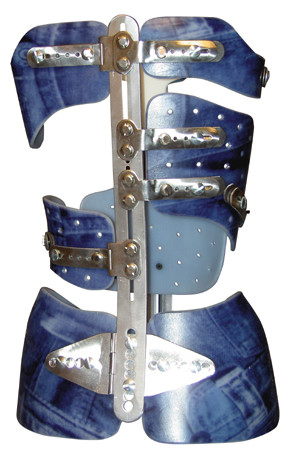
**Lyon soft polyethylene brace**.

For cases of neurological scoliosis, wheel chair dependent patients are fit with the soft Lyon Brace in high or low density polyethylene (Figure 19).

## Contraindications to brace wear

Noted contraindications include:

- Juvenile and infantile scoliosis in order to avoid a tubular thorax

- Severe thoracic lordosis for which the treatment is usually surgical. However a temporary brace before surgery can be applied with a Lyon brace focalized on the lumbar curve to limit the fusion of the lower lumbar vertebrae.

- Major psychological reactions.

- Non acceptation of the plaster cast. The conservative treatment from Lyon is very elitist compared to other treatments. When the child and family accept the plastered brace, compliance is maximal.

### Prescription of the Lyon Brace

There is an optimal brace blue print and design that matches each of the 14 curve patterns identified in Lenke's classification [3,5].

**Figure 20:**
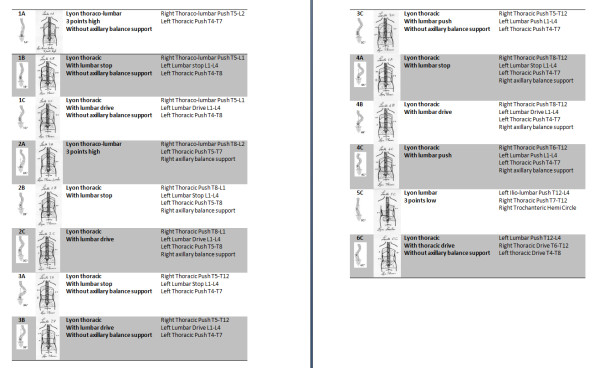
**Bueprints**. corresponding to 14 types of the Lenke's classification for a right thoracic and left lumbar scoliosis

They are summed up for right thoracic and left lumbar curves (Figure 20).

### The Fitting protocol

The plaster cast application and subsequently the brace fitting procedure are accomplished in the out-patient clinic [6].

The protocol depends on the magnitude of the Cobb angle and is summed up in (Table 1).

**Table 1:** Protocol of the Lyon Brace

Cobb Magnitude	Nb and time in the plaster cast	Wearing of the Lyon brace	Time to begin Weaning
**< 30°**	1 cast -1 month	nightly	End of height growth

**30-40°**	2 casts - 1 month each	After school 16 h/24	1 year after the end of height growth

**> 40°**	2 casts - 2 months each	23 h/24	2 years after the end of height growth

The protocol depends on the angle of the scoliosis

Physiotherapy is performed twice a week when the patient is in the plaster cast and once per week while wearing the brace. After removal of the brace the patient can participate in sporting activities as much as is desired.

### Physiotherapy treatment principles

- No complex instructions or difficult procedures. All the exercises have to be repeatable at home.

- All sporting activities are encouraged and should be continued as long as they are not contraindicated. Some movements may need to be adapted to avoid undesirable body positions like deep, quick inspiration and forward trunk flexion.

- The exercises must be symmetric in the frontal plane.

- No chapel and miracle exercise. Choosing the best technical approach for each child, at every age, and every therapeutic sequence is crucial.

- Compliance, not defiance, is promoted through an evolution in the exercises which are repeated for a few minutes a day at home [7,8].

Treatment is focused on the whole child and not on the x-ray or scoliosis curve alone. Assessing the child's physical appearance is fundamental; for example, asymmetry with plagiocephaly may be an indicator of juvenile scoliosis. However; it is equally important to assess the child's emotional appearance observing the expression of the patient's moods. Do they express worry, fear, anxiety, despair, indifference, disappointment and/or aggressiveness? It is our role to change this look to achieve a relationship based on reliance and complicity (for more details see Additional file 1).

## Results

This study presents the results of 1338 subjects diagnosed with scoliosis and treated in France and Italy according to the above indications. All the assessments were done by the same physician (JCdM) and the data was entered into the Excel table by his secretary during the "check-up", two years after brace weaning. All subjects previously treated with the Milwaukee brace are included. All bracing failures, with progression towards surgery, are included. Every subject not checked at least two years after the weaning of the brace is excluded. Considering our recruitment, only 8 cases were non-idiopathic: 6 cases diagnosed with Down syndrome and 2 cases with Neurofibromatosis. Finally, the 12 patients with paralytic scoliosis were treated with a Soft Lyon brace and are excluded from the study.

The evaluation criteria are classical. Most subjects were female. The mean age was 13 years and 10 months (+-1.7) at the beginning of treatment. Standing height was 159.81 cm (+-11); weight 47.04 Kg (+-9), and vital capacity 2.20 l (+-7). Two years after the weaning of the Lyon Brace, her height was 165.00 (+-6.7) cm, her weight 54.85 (+-8.17) Kg, and her vital capacity 2.63 (+-6.2).

All the cases are grouped according to the type of Lyon Brace prescribed. The results are expressed in Cobb angle measurement and in percentage of reduction according to the initial angulation in (Table 2).

**Table 2:** Angular results in Cobb's degree and percentage of reduction compared to the initial angulation

1338 cases	Cobb initial	Cobb plaster	Cobb Brace	Cobb weaning	2 years after
Lyon Thoracic	33.28 (+-8)	10.98 (+-8.53)	15.09 (+-9.16)	27.52 (+-10.62)	29.29 (+11.65)
(thoracic)		**67%**			**12%**
285 cases					

Lyon Thoracic	31.45(+-8.88)	12.39(+-8.25)	15.78(+-9.17)	27.15(+-10.52)	27.92(+-10.68)
(double major)	28.81(+-8.14)	9.27(+-7.40)	11.66(+-6.81)	22.16(+-8.60)	21.70(+-8.69)
351 cases		**61%/68%**			**10%/25%**

Lyon Thoraco	28.54(+-6.98)	5.65(+-6.62)	9.41(+-7.76)	20.64(+-8.99)	21.81(+-9.68)
Lumbar		**80%**			**24%**
279 cases					

Lyon Lumbar	27.97(+-6.33)	6.07(+-5.79)	7.24(+-6.50)	17.45(+-7.66)	17.92(+-7.83)
450 cases		**78%**			**36%**

All the cases of scoliosis are grouped according to the type of Lyon Brace applied. The results are expressed in Cobb angle and in percentage of reduction according to the initial angulation.

The aesthetic results at the rib hump level (Rib H) measured in mm are grouped together (Table 3).

**Table 3:** Results in mm about Rib Hump

1338 cases	Rib H initial	Rib H Corset	Rib H weaning	2 years after
Lyon Thoracic (thoracic) 285 cases	25.43 (+-8.68)	10.12 (+-7.52)	15.54 (+-8.39)	17.03 (+8.70) **33%**

Lyon Thoracic (double major) 351 cases	19.32(+-9.61) 13.82(+-7.32)	7.55(+-5.86) 2.69(+-3.12)	11.96(+-7.13) 6.19(+-4.39)	13.21(+-7.71) 6.92(+-5.45) **32%/50%**

Lyon Thoraco-Lumbar 279 cases	21.72(+-7.31)	6.20(+-5.35)	11.23(+-5.73)	11.62(+-6.74) **46%**

Lyon Lumbar 450 cases	17.42(+-7.37)	2.55(+-2.70)	5.59(+-4.42)	6.33(+-5.43) **64%**

In this time, in France, the Bunnel's ATR was not used.

In accordance with international norms, the results, two years after the weaning of the brace, were divided into three groups:

- Good, i.e. an improvement of more than 5° compared to the initial curve;

- Stable, i.e. +- 5° compared to the initial curve;

- Failure, i.e. a progression of more than 5° compared to the initial curve.

For double major scoliosis, half the sum of the two curves is compared.

The results are presented in (Table 4).

**Table 4:** Results at +- 5°

	Improvement >5°	Stable +-5°	Progression > 5°
Lyon Thoracic (thoracic) 285 cases	140 54.26%	85 32.95%	33 12.79%

Lyon Thoracic (double major) 351 cases	208 59.26%	127 36.18%	16 4.57%

Lyon Thoraco-Lumbar 279 cases	189 67.74%	76 27.24%	14 5.02%

Lyon Lumbar 450 cases	362 80.44%	84 18.66%	4 0.88%

Total 1338 cases	899 67.19%	372 27.80%	67 5.00%

- Good, i.e. an improvement of more than 5° according to the initial curve

- Stable, i.e. +- 5° according to the initial curve

- Failure, i.e. a progression of more than 5° according to the initial curve For double major scoliosis, half the sum of the two curves is compared.

A sub-group of 174 scoliosis subjects whose treatment was started at Risser 0 were isolated and analyzed. The results are obtained comparing the average of the main and secondary curves (Table 5).

**Table 5:** Angular results in Cobb's degree and percentage of reduction compared to the initial angulation.

	Cobb initial	Cobb plaster	Cobb Brace	Cobb weaning	2 years after
Total cases 1338 cases	28.51 (+-7.14)	8.09 (+-7.16) **72%**	10.75 (+-7.69)	21.27 (+-9.09)	21.80 (+9.48) **24%**

Group Risser 0 174 cases	27.23(+-6.46)	5.24(+-6.65) **81%**	8.85(+-6.88)	21.85(+-8.98)	22.31(+-10.22) **18%**

Group of 174 subjects with juvenile scoliosis in which treatments were initialized at Risser 0. Comparions made with the global statistics.

If treatment was started when the patient had an angulation less than 40° then only 2% of the patients required surgery. For angulations greater than 40° at the beginning of the treatment, the percentage progressing to surgery was 20%. The indication for surgery depends on the stability of the scoliosis at the end of treatment.

The 2 groups, characterised by an improvement above 5°, stability at +- 5° and a progression of more than 5°, were studied (Table 6).

**Table 6:** Results at +- 5°

	Improved >5°	Stable +-5°	Progressed > 5°
Total cases (1338 cases)	899 67.19%	372 27.80%	67 5.00%

Group Risser 0 (174 cases)	85 48.89%	54 31.11%	35 20%

Study of the 2 groups according to an improvement above 5°, stability at +- 5° and a progression of more than 5°.

## Discussion

The results of the Lyon Brace Management have been validated by numerous teams in Europe [9,10]. However, the plaster cast process is quite involved for the patient and physician. Consequently, many teams looked for more convenient and expedient solutions without preliminary plaster cast reduction [11]. The results are debatable [12].

The Lyon Braces have continued to improve with the progress of technology while adhering to fundamental biomechanical principles. For 20 years, it has been consistently applied and evaluated in several public and private centers in both France and Italy. The results remained the same during successive studies and the variations in time are linked to the screening and an initial angulation which was lower.

The curve reduction and improved spinal alignment achieved in the plaster cast facilitates a better moulding on a more symmetrical and balanced spine. Using his expertise and the x-ray in the plaster cast as a reference, the CPO fabricates and fits the patient with the brace. This reduction enables a night bracing, which is greatly appreciated by the child, when the curve is below 30° (Table 1).

The Lenke curve classification system was found to be most beneficial in helping the physician classify the curve, prescribe the most appropriate Lyon Brace and guide the CPO in constructing it.

The ideal patient population indicated for treatment with the Lyon Brace are adolescents at the age of pubertal growth. They are less susceptible to the corrective forces that can cause a chest wall deformity or tubular thorax in younger juvenile patients.

We do not use only the Lyon brace for AIS, but its adaptability to all types of curvatures is great.

To meet study inclusion criteria the initial angulation is always the one before the plaster cast, even if the patient was previously treated.

The application of a plaster cast at the beginning of the treatment may be a way to select the most motivated patients for whom compliance is the best. In the same way, many "drop-outs" are noted at the end of the treatment and only the motivated ones come to the check-up. These "drop-outs" are not part of the 1338 cases reported.

If a patient eventually undergoes surgery, he is included in the statistics as a bracing failure, and the pre-surgical curve angulation is used for statistical calculations.

The important Standard Deviation of the initial Cobb's angle of our series shows the great range of angular indication, from the weaker curve to the most important. The only variable is going to be the time of wearing the brace.

In evaluating post treatment outcomes, the best results were obtained for lumbar curvatures. The brace is short and well tolerated; unfortunately, these curves are often painful during adulthood and evolve towards rotary dislocation.

The double major curves respond well to the Lyon Brace, despite the short lever arm in the frontal plane.

Similarly, the thoraco lumbar curves respond well to the Lyon type III brace. The excellent lever arm effectively maintains the good correction achieved from the plaster cast. Nevertheless, it is this group that most often progresses towards surgery, even when the treatment is followed acutely. Therefore, strict brace wear is critical, despite the sometimes impressive initial corrections obtained in the plaster cast.

When examining the rotational changes effected by the brace, the rib hump is often better corrected than the angulation. It is frequently reduced by 33% at the thoracic level and more than 50% at the lumbar level. The aesthetic improvement is always better than the X ray appears.

The excellent global index of effectiveness of all the curves is 0.95, and it can be explained by the selection of the patients. The index is only 0.87 for the thoracic curves. The most relevant subjects to judge the effectiveness of the Lyon Brace is the Risser 0 group, of which the index of effectiveness for the most progressive scoliosis is 0.80.

But, if the mean curves of the Risser 0 group are compared with the mean curves of the general statistics, the two curves are almost the same, as if the Lyon Brace was stopping the scoliotic curves whatever the age of the child.

Even if the index of effectiveness seems to be better, it is very hard to compare the results of the Lyon Brace study with any of the other published brace studies. This is, in large part, due to the Lyon treatment protocol requiring plaster casting and physiotherapy. The effectiveness of therapy associated with bracing having been previously established in work by Négrini [13].

Additionally, adhering to the main treatment indication which is adolescents at pubertal growth, may also help explain the good results.

## Conclusions

The Lyon Brace management is a very effective conservative orthopaedic treatment which is conclusively demonstrated by the long-term results presented in this study. The out-patient clinic application of the plaster cast and the Lyon Brace is more cost effective than in-patient hospitalization and enables the child to continue going to school.

The physician, using the Lenke classification system, can easily identify the curve pattern and consistently prescribe one of the three brace types. The adaptability of the design and the expertise of the CPO allow the patient to be fit with a comfortable, adjustable, and effective Lyon Brace.

It is a complement and an alternative to other braces and must be included in the therapeutic range of the scoliosis specialist.

This treatment approach is one that is most likely to be successful for the child as well as for the physician.

## Consent statement

Written informed consent was obtained from the patient for publication of this research and accompanying images. A copy of the written consent is available for review by the Editor-in-Chief of this journal.

## Competing interests

The authors declare that they have no competing interests. The use of Lyon Brace is totally free without patent fees. The metallic kits are available at numerous suppliers.

## Authors' contributions

JCdM designed the study and carried out the medical literature research and drafted the manuscript; CL carried out the technical literature research. For more than 20 years, he leads the technical development of the Lyon brace. FB participated in drafting and revising the manuscript. All authors read and approved the final manuscript.

## Supplementary Material

Additional file 1 **Physiotherapy during bracing - The Lyon Method for physiotherapy was first published in 1978 **[7] Click here for file
