# Supplementary material for: "Brace Technology" Thematic Series - The Lyon approach to the conservative treatment of scoliosis
Source: Scoliosis. 2011 Mar 20;6:4. doi: 10.1186/1748-7161-6-4 (PMC3069938; doi:10.1186/1748-7161-6-4)
Supplement: Additional file 1 — Physiotherapy during bracing - The Lyon Method for physiotherapy was first published in 1978 [7] [file 1748-7161-6-4-S1.PDF]

Appendix 1

# Physiotherapy during the Lyon Brace treatment

| OBJECTIVES | EXERCISE | DANGER-CARE |
|---|---|---|
| Modification of the spoiled body image of the scoliotic | When we first see the patient, back and vertical height difference pictures are shown to the child<br>He has to be conscious of the deformity thanks to the mirror or a video tape | The cortical representation of the back is weak and damaged by the fast growth, but let's be careful not to devaluate and depreciate the image of the body |
| Supplying up of the retracted elements of the concavity | Elongation posture (Mézières, RPG) | The rigidifying scoliosis curve may be a natural element of stability. An excess of the supplying up can lead to a progressive revival of the scoliosis. |
| | Dynamical mobilisation | In some double curves case, the mobilization is the same on the right and on the left, in fact the bendings show us that 80% of the movement happen in the correction sense and 20% in the worsening sense. |
| | Manual modelling of the vertical height difference | Be careful not to favour the empty back. The support has to happen on the internal side of the vertical height difference. A cushion is put under the left chondro costal canopy and the transversal movement leads to exhalation. |
| Suppling up of the griddle | Segmentar and analytical correction of the deficit of the extension of the hip measured by Biot at 43%, since the youngest time of the patient | The belts have to compensate |
| Improvement of the vital capacity | Highering of the VEMS<br>Blow a balloon every night | The deep inspiration favours the rotation (Geyer) thus slow inspiration and quick exhalation. |
| Saving of the spine<br>Diminution of the mechanical constraint on the axis | Development of the compensation at the belt level and the membres on a trunk which is still close to vertical | |
| Reharmonisation of the static | Repositioning of the head on the gravity line in the frontal and sagittal plane. Exercice to carry big charges. We look for the global balance, the sand pack must stay on the head, the harmony and the movement coordination. The walk must be synchronised with breathing. For lumbar scoliosis and thoraco-lumbar : learning of the shift. | A pelvis unbalance or of the scapular belt can compensate a scoliosis, we have to respect them.<br>All types « C » of Lenke, the opening of the illio-lumbar angle goes in the direction of the accentuation of the lumbar curve. In the sagittal plane,we have to avoid favouring the lordosing vertebral rear. |
| Strengthening of the muscle in order to make the behavior in a corrected position easier | Reinforcing of the fibres of the deep paravertebral muscle structure and muscles stabilizing the belt such as the psoas, the abdominals and the pectorals by | « The brain ignore the muscles and only know the movement »<br>In the frontal plane, The exercices are symmetric because we do not know the role of the |

| | | |
|---|---|---|
| | powerful slow static contraction supported in a corrected position. | asymmetry concavity convexity.<br>In the sagittal plane, the anterior flexion increase the rotation, therefore we have to strengthen in a neutral position. No body building which concerns the superficial muscle structure. |
| The 24 hours of the back : adaptation of the scoliosis to the environment and of the environment to the scoliosis | Control of the sitting position when listening and writing according to the morphotype<br>Dealing with the school bag | |
| Stimulate the maturation and the balancing postural system | Proprioceptiv rehabilitation kyphotisation from :<br>Feet sensors,<br>ocular sensors,<br>cutaneous sensors | Some patients have a postural reflex when there is an unbalance situation, which leads to a worsening of the scoliosis |
| Physiological valorisation<br>Stimulate the global mobilisation of the spine in an automatic way | Sport practice<br>Coordination of the gesture, harmony of the move | |
| Psychological valorisation<br>Well being and self confidence | To be upbeat with the scoliosis<br>« The scoliosis is not a disease but a symptom» | |
